# Role of noble and rare earth metals in bioactive materials for medical applications in tissue engineering

**DOI:** 10.1039/d5ra04036a

**Published:** 2025-10-27

**Authors:** Katarzyna Staszak, Martyna Rzelewska-Piekut, Magdalena Regel-Rosocka

**Affiliations:** a Institute of Chemical Technology and Engineering, Faculty of Chemical Technology, Poznan University of Technology ul. Berdychowo 4 60-965 Poznan Poland magdalena.regel-rosocka@put.poznan.pl

## Abstract

Tissue engineering (TE) is an interdisciplinary field that is developing rapidly. It combines medicine, chemistry, and biology to create functional biological materials that can restore or replace damaged tissues and organs. However, the potential of bioactive metals to improve the mechanical properties of tissue engineering scaffolds is largely unexplored. Our review of recent literature, focusing primarily on research conducted between 2020 and 2025, reveals common applications of these two metal groups, including bone and soft tissue regeneration, wound healing, and anticancer therapy. It also reveals differences in their use, highlighting the abundance of rare earth elements (REE) applications in the medical field for imaging and diagnostics, medical lasers, and radiation shielding and protection. The role of REEs in drug delivery systems distinguishes them from noble metals in medical applications. Here, we demonstrate that noble metals and REEs can significantly enhance the strength and fracture resistance of tissue engineering scaffolds. In the form of nanoparticles, these metals enhance biocompatibility, ensuring seamless integration with biological tissues and reducing the risk of adverse reactions. Key findings from the literature review highlight future directions for noble metal and REE applications in TE and medicine, including the multifunctionality of new biomaterials (particularly REE biomaterials), smart nanomaterials that respond to various changes in organisms, hydroxyapatite (HAP) doped with REEs, and surface modifications (particularly Pt-, Au- and Ag-doped materials) to create multifunctional implants that can better integrate with biological tissues and reduce infection risks.

## Abstract 2

An overview of the potential and limitations of the role of noble metals and rare earth elements as bioactive metals in medical applications and regenerative therapies – from antimicrobial treatments to drug delivery systems.

## Graphical abstract

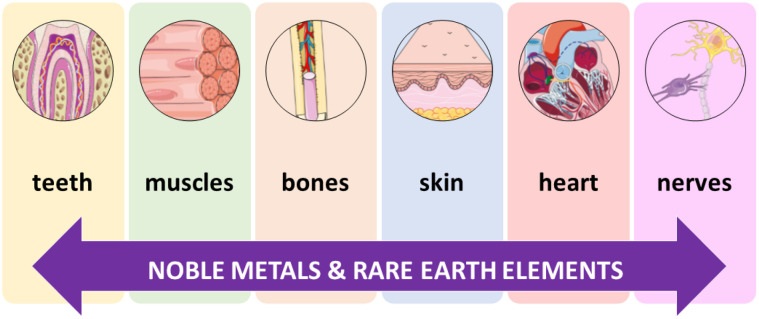

## 1 Introduction

The primary objective of bioinorganic chemistry is to explore the biological properties and bioactivity of metals and their complexes, which could potentially be used in medicine, for example. The discovery of cisplatin, an inorganic Pt(ii) complex that functions as an anticancer drug, was an essential moment that spurred extensive research in the field of bioinorganic chemistry. This breakthrough highlighted the cytotoxic potential of various organometallic compounds to target specific cancer cells and suggesting their potential use of these compounds in chemotherapy.^1^ Concurrently, biomaterials used in a variety of medical applications typically comprise metals and alloys, carbon materials, polymers, ceramics, and composites. For example, rare earth elements (REEs) have drawn attention due to their ability to enhance osteogenic differentiation, stimulate mineralization, and modulate cellular responses, making them promising candidates for bone tissue engineering applications. The incorporation of REEs into scaffolds has been explored as a strategy to enhance bone repair outcomes.^2^ It should be mentioned that the biological and mechanical properties of scaffolds depend on the metals doped, and the method of scaffold preparation plays an important role in determining the material's performance in tissue engineering. Techniques for fabricating scaffolds can be classified based on various criteria, including the method of material processing, the type of materials used, and the intended application. Among these techniques are often used to manufacture metal-based biomaterials bottom-up techniques including sol–gel synthesis, electrospinning, 3D bioprinting, and freeze-drying, and top-down techniques such as melt-quenching or melt-moulding.^3–8^

**Fig. 1:**
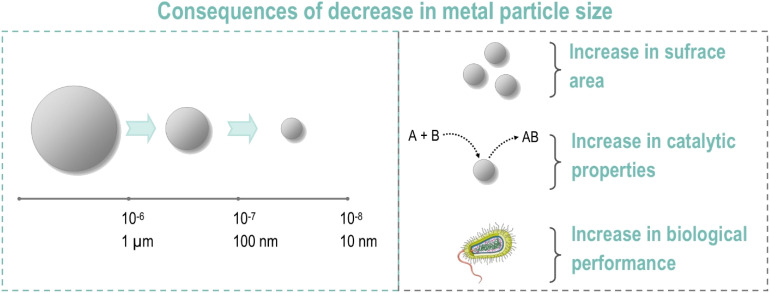
Crucial properties of nanoparticles of metals in biomaterials.^9^

Many substances exhibit novel and intriguing properties when they are in a nano form, in comparison to their counterparts with larger particle diameters (*e.g.* micro). To illustrate, in the case of metals (especially platinum group metals (PGMs)), a decrease in particle size (μm → nm) is accompanied by an increase in surface area and reactivity, which enhances their catalytic and biological performance (Fig. 1).^9^

NPs can be formed from a variety of chemical structures, including metals, metal oxides, silicon compounds, ceramic materials, organic and biological particles. These diverse structures enable the development of novel functionalities that differ from those of their micro-sized counterparts. For example, silver nanoparticles exhibit enhanced antimicrobial activity compared to bulk silver,^10^ gold nanoparticles show unique optical properties such as surface plasmon resonance useful in imaging and diagnostics.^11^ NPs are used in almost every area of the human life, spanning from domestic products and pharmaceuticals to the chemical industry and numerous medical specialities (including orthopaedics, dentistry, medical imaging, cardiology, wound healing, and neural implantation). For example, in orthopedics, silver nanoparticles (AgNPs) are incorporated into bone implants and coatings for their antimicrobial properties, effectively reducing infection risks and promoting osseointegration.^12,13^ Additionally, cerium nanoparticles (CeNPs) have demonstrated bioactivity and biocompatibility, making them promising materials for bone regeneration scaffolds.^14^ In dentistry, gold nanoparticles (AuNPs) are utilised in restorative materials to enhance mechanical properties and provide antibacterial effects, contributing to improved dental care outcomes.^15^ In medical imaging, gadolinium-based compounds such as gadolinium oxide nanoparticles (Gd_2_O_3_NPs) are proposed as contrast agents in magnetic resonance imaging (MRI), providing enhanced imaging capabilities for accurate diagnosis.^16^ In cardiology, platinum-based materials are not only used in stents for their biocompatibility and corrosion resistance, but recent studies have also highlighted the therapeutic potential of platinum nanoparticles (PtNPs) in treating atherosclerosis. For instance, Pan *et al.*^17^ developed a microfluidic model that mimics the AS microenvironment, including key factors like fluid shear stress and cyclic stretch. Their findings demonstrated that this pathological environment significantly damages endothelial cells and cardiomyocytes. For wound healing, silver nanoparticles are integrated into dressings to expedite tissue regeneration and prevent microbial infections, leveraging Ag potent antimicrobial properties.^18^ In neural implantation, gold and platinum nanoparticles are explored to enhance the electrical conductivity and biocompatibility of neural interfaces, facilitating better signal transmission and integration with neural tissues.^19^

Despite their promising applications, concerns persist regarding their long-term safety and environmental impact of metal nanoparticles. Toxicity and degradation issues present challenges to the widespread adoption of these materials in biomedical contexts, influencing both the cost and accessibility of noble metals and REEs.^20^ It is essential to address these limitations and develop innovative strategies to overcome them in order to advance the practical use of noble metal and REE biomaterials in various medical applications.

Among many reviews on metals in tissue engineering and medicine, especially the newest ones, most authors focus on a single metal or one group of metals, as well as one medical application for the group of metals.^21–25^ Therefore, our intention in focusing on original research papers is to provide a fresh perspective on the subject, rather than reiterating previously published reviews. In this review, ‘bioactivity’ is defined as ‘any beneficial or adverse effect that an artificial substance has on living matter’.^26^ This includes exploring the use of noble and REE in tissue engineering, examining their unique properties in various forms, such as nanoparticles, and assessing their potential benefits and challenges. This review highlights how these metals can enhance the mechanical properties and biocompatibility of materials used in medical applications, ranging from antimicrobial treatments to drug delivery systems. The work aims to provide a comprehensive overview of the latest advances and applications of two groups of metals – noble metals and REEs – in bioactive materials for medical applications, with a particular focus on the period from 2020 to 2025, as evidenced by recent literature. Additionally, it highlights the potential and limitations of metal-based biomaterials in advancing medical and regenerative therapies.

## 2 Noble metals

Noble metals including Au, Ag, and the platinum group metals (PGMs: Pt, Pd, Rh, Ru, Ir, and Os) are among the most desirable metals in the world, due to their high chemical and thermal resistance, ability to catalyse chemical reactions, corrosion resistance, high conductivity, density, and melting and boiling points. For this reason, PGMs can be used in various areas, including medical applications. Platinum group metals, based on their atomic structure, are divided into light PGMs (Ru, Rh, and Pd) and heavy PGMs (Pt, Ir, and Os). The ionic forms of PGMs have not only positive biological activity (promising anticancer activity) but also biotoxicity (to healthy cells) due to their unique ability to coordinate or react with various bioactive molecules.^27^

**Fig. 2:**
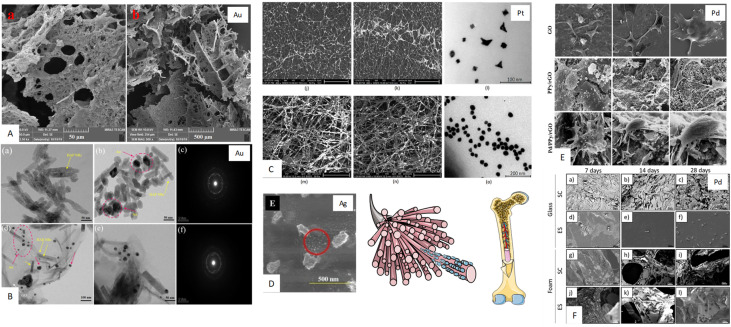
Noble metal-based materials in bone tissue engineering: (A) scanning electron microscopy (SEM) images of collagen/CNCs/CS-Au hydrogels (scale bar: (a) 50 μm and (b) 500 μm). Collagen/CNCs/CS-Au: collagen/nanocrystalline cellulose/chitosan loaded with Au nanoparticles. Reprinted from ref. 41. (B) TEM image of (a) HAP, (b) HAP/Au, (c) SAED Pattern of HAP/Au, (d) and (e) GO/HAP/Au, (f) SAED Pattern of GO/HAP/Au nanocomposite. Reprinted from ref. 40. (C) (j) SEM microphotograph of the PLA nanofibers obtained under 30 kV covered with Pt nanoparticles (scale bar 50 μm); (k) SEM microphotograph of the PLA nanofibers obtained under 35 kV covered with Pt nanoparticles (scale bar 50 μm); (l) TEM microphotograph of the prepared Pt nanoparticles; (m) SEM microphotograph of the PLA nanofibers obtained under 30 kV covered with Au nanoparticles (scale bar 30 μm); (n) SEM microphotograph of the PLA nanofibers obtained under 35 kV covered with Au nanoparticles (scale bar 30 μm); (o) TEM microphotograph of the Au nanoparticles. Reprinted from ref. 38. (D) and (E) High magnified SEM image of CS–Ag–1.5 M scaffold depicting the presence of silver nanoparticles indicated by red circle. Reprinted from ref. 29. (E) Osteo cell attachment studies: FE-SEM images of Saos-2 osteo cells cultured on GO, PPy/rGO, and Pd/PPy/rGO NC with diverse magnification. Reprinted from ref. 37. (F) SEM images of human Saos-2 cells cultured for 7, 14 and 28 days under standard conditions (SC) or electrical stimulation (ES) on either glass coverslips (a–f) or Pd-coated PU foams (g–l). Reprinted from ref. 68. (Free images from Smart Servier Medical Art, website: https://www.smart.servier.com).

What makes noble metals such as Ag, Au, Pt, Pd highly suitable for tissue engineering applications, especially in bone TE (Fig. 2), are their unique properties which have been highlighted by many researchers.^28–31^ The crucial characteristics of TE are as follows: excellent biocompatibility, meaning they do not induce adverse reactions when implanted in the body, high resistance to corrosion and oxidation, even at elevated temperatures, ensuring their stability and longevity in biological environments, good electrical conductivity which positively influence cell attachment and proliferation properties; and unique optical properties due to surface plasmon resonance (SPR). This property is particularly useful for bioimaging and diagnostic applications, as it enhances the functionality of tissue engineering scaffolds. Noble metals are used in bioactive materials in the form of NPs due to their nanometric size, large surface area, and the number of particles per unit of mass compared to their larger counterparts. They can also be modified in terms of shape and size, and can be surface functionalised. Examples of the main applications of noble metals in TE are presented in Table 1.

**Table 1:** Noble metal-based materials in tissue engineering^a^

Application	Metal and material characteristics	Ref.
Bone regeneration	**Ruthenium**	32–34
	• 3D printed Ru-loaded PEGylated liposome scaffold (drug delivery system) for osteosarcoma inhibition and bone TE (confirmed *in vivo*); proper for implantation into bone defect sites after surgery and a good antitumor effect over a relatively long period	
	• Ru red as a TRPV5 channel inhibitor; regulates osteoclast degradation of artificial bone	
	**Palladium**	35–37
	• PdNPs on reduced graphene oxide scaffold → antibacterial, bone implant potential	
	• PdNPs nanofunctionalised HAP scaffolds → marrow stem cell proliferation	
	• Fe–Pd-bredigite biocomposites (SLM) as biodegradable implants for bone tissue regeneration/organ repair → high biodegradation, bioactivity, cytocompatibility, mechanical strength	
	**Gold**	38–41
	• Novel hybrid 3D PLA/chitosan scaffolds with AuNPs → osteogenic, antibacterial, biocompatible, potential substitute for BMP. Gelatine–Au hydrogels were effective in rabbit bone TE (*in vivo* study)	
	• AuNPs in *in situ* chitosan-based hydrogels (Schiff base crosslinking) → stronger mechanics, better degradation resistance, simplicity in tailoring to provide tissue-specific networks for nerve, cardiac, bone tissues	
	**Platinum**: PLA/chitosan scaffolds doped with PtNPs → enhanced bone regeneration	38
	**Silver**: Metallic Ag, Ag salts, and AgNPs in scaffolds (cryogelation, electrospinning, freeze–thaw) → antibacterial, antiviral, mechanical reinforcement	33 and 42–46
Wound healing	**Ruthenium**: One-step fabrication of biomineralized tough hydrogels (BTHs) *via* visible-light mediated nano-biomineralization with Ru-based redox photochemistry; injectable or 3D-printable complex structures for the repair of specific wounds	47
	**Iridium**: IrNPs (PVP-coated) simulate antioxidant enzymes (smart nanomaterials) → promote wound healing, ROS elimination	48
	**Gold**: AuNP incorporating thermoresponsive Pluronic®127 or hydroxypropyl methylcellulose gels (smart materials) → promote wound healing, antibacterial activity	49 and 50
Soft tissue regeneration	**Palladium**	36 and 51
	• Pd–Cu–Ni–P metallic glass wires in gelatin methacryloyl gels for skeletal muscle regeneration → high conductivity, mechanical performance	
	• Fe–Pd-bredigite biocomposites (SLM) as biodegradable implants for bone tissue regeneration/organ repair → high biodegradation, bioactivity, cytocompatibility, mechanical strength	
	**Gold**: AuNPs in electrospun chitosan-PCL conductive scaffolds → improved electrical signal transfer	44 and 52
	**Iridium**	53–55
	• IrOxNPs as corona neural interfaces → enhanced neuronal activity, miniaturized neural implants	
	• IrNPs-PVP artificial enzymes (antioxidant, biocompatible) → treatment of acute kidney injury	
	**Silver**: AgNPs–agarose–chitosan hydrogels and AgNPs–polyaniline aerogels → biocompatible, antibacterial scaffolds	56 and 57
Anticancer therapy	**Gold**	58 and 59
	• Au(iii) α-*N*-heterocyclic thiosemicarbazone compounds as a new gold-based agent targeting gastric cancer cells → enhanced bioavailability and inhibition gastric tumor growth (*in vivo*)	
	• Au(i)–phosphine complexes cytotoxic → stability and optimal lipophilicity of the complexes resulting in high cytotoxicity against ovarian cancer cells, even higher than cisplatin	
	**Platinum**: Pt(ii) pyrazoles/thiazoline and thiazine complexes → enhanced cytotoxicity against cervical and colon cancer cells	27 and 60
	**Palladium**: Bioactive HNP–Pd complex (HNP = 1-(pyrimidin-2-yliminomethyl)-naphthalene-2-ol) → significant antimicrobial activity, inhibits cancer cell growth	61
Dentistry	**Platinum**: Pt alloys → biocompatible, corrosion resistant, non-allergenic in saliva	45
	**Rhodium**: Rh-coated stainless steel & NiTi alloys → rougher surface, higher hardness, lower friction in orthodontics	62–64
Implants, scaffolds, and cardiac	**Gold**	41, 44, 52, 65 and 66
	• AuNPs (collagen/CNCs/CS-Au) → improved mechanical strength and degradation resistance of the scaffolds	
	• Microwrinkles on polymer cantilevers guide cardiomyocyte growth; Au nanospheres/rods/wires improve conductivity in cardiac patches post-MI.	
	**Platinum**: Pt stents (corrosion-resistant, stable, biocompatible); Pt – an ideal electrode material for cardiac pacemakers	45
	**Silver**: Graphene-Ag/polyurethane scaffolds (electrospinning) → improved conductivity & mechanics for cardiac tissue repair	46 and 67

a CNC – nanocrystalline cellulose, CS – chitosan, HAP – hydroxyapatite, NP – nanoparticles, PVP – poly(vinylpyrrolidone), ROS – reactive oxygen species, TE – tissue engineering.

### 2.1 Bone regeneration

Bone tissue is highly organised and mostly composed of inorganic components. The latest generation of scaffolds is expected not only to be biocompatible, but also to promote osteoconduction. Therefore, chitosan is commonly used as a biopolymer that positively affects the behaviour of osteoblasts. Various metallic NPs and other additives are incorporated to influence the material properties (Fig. 2).

For example, in bone TE, AuNPs optimise stem cells, and improve scaffold performance.^69^ This is a great opportunity to design innovative feasible hydrogels composed of collagen, aldehyde modified nanocrystalline cellulose, and chitosan loaded with gold nanoparticles (collagen/ADH-CNCs/CS-Au). Depending on the molar ratio of the hydrogel components, the microscopic morphology (Fig. 2A), equilibrium swelling, *in vitro* degradation, and mechanical properties were significantly affected. AuNPs supported thermal stability and cell proliferation due to intercellular communication through the electrical conductivity of gold. Moreover, these collagen/ADH-CNCs/CS-Au hydrogels exhibit no cytotoxicity towards the NIH 3T3 cell line and showed a great potential in developing tailored tissue-specific networks, not only for bone regeneration, but also for nerve, and cardiac tissues.^41^

In turn, the addition of Ag and CuNPs enhances the antibacterial properties of bioactive glass (BG), which is useful for coating medical sutures. Ag-BG is more effective as a bactericidal agent than Cu-BG and can be used in bone regeneration.^70^ Furthermore, doping Au, Pt and TiO_2_ into novel hybrid 3D scaffolds obtained through PLA electrospinning and microwave-assisted chitosan crosslinking produced materials with high porosity, excellent swelling properties and the ability to undergo biomineralisation electrostimulation. The distribution of metallic NPs in the matrix differs and influences the properties of these biomaterials. Unlike TiO_2_ and PtNPs, AuNPs are present on the surface of the fibrous layer and in deeper layers (Fig. 2C). In terms of bioactivity upon contact with cells, the highest was exhibited by the matrix modified with nanoHAP and amorphous TiO_2_NPs, while the highest positive impact on DC-stimulated *in vitro* biomineralisation was shown by the scaffolds containing AuNPs, which makes them prospective for bone-tissue regeneration.^38^ Similarly, AuNPs were uniformly dispersed in the GO(graphene oxide)/HAP/Au ternary nanocomposite incorporated into the polymeric film of chitosan and polyvinyl alcohol (Fig. 2B). This material exhibits good antibacterial properties and improved osteoblast cell viability, making it potentially applicable to bone tissue regeneration.^40^

PdNPs, when incorporated into HAP or GO scaffolds, improve mechanical strength, antibacterial performance, biocompatibility, and osteoproliferation, thus, offering promise for bone tissue regeneration.^35,37,71^ Calabrese *et al.*^35^ proposed nanofunctionalised HAP scaffolds with Au nanorods or PdNPs for bone regeneration, which enhance stem cell proliferation. Heidari *et al.*^71^ modified the surface of the HAP scaffolds with ZnO and Pd, improving their mechanical properties (the compressive strength and fracture toughness) and antibacterial properties. Murugesan *et al.*^37^ proposed reduced GO as a scaffold with PdNPs for bone tissue (Fig. 2E), which can improve the antibacterial properties and make it a promising material for bone implants. Another important feature of biocompatible metallic scaffolds is their intrinsic conductivity, making them ideal candidates for electrical stimulation (ES). Pd-coated polyurethane foams were proven to enhance by ES proliferation of osteoblasts (the Saos-2 cell type) and upregulate expression genes related to extracellular matrix formation (Fig. 2F).^68^

Besides Pd, the role of other PGMs in bone scaffolds has been indicated, but to a much smaller extent. Organometallic complexes of Ir(iii) have been reported to inhibit bacterial growth, including resistant strains such as *S. aureus*.^72,73^ Whereas, a Ru-labelled polysaccharide-based hydrogel has been proposed for bone healing (test studies have been conducted on rabbit bone defects).^74^

Overall, especially Au and PdNPs are the key enhancers of scaffold performance for bone regeneration through improved bioactivity, antibacterial function, mechanical integrity, and responsiveness to electrical stimulation.

### 2.2 Wound healing and soft tissue regeneration

Reactive oxygen species (ROS) play a crucial role in wound healing. However, excessive levels of ROS are reported to induce DNA damage, and changes to protein structure and lipid peroxidation (MDA), as well as reducing the glutathione-oxidized glutathione ratio (GSH/GSSG), which impairs wound healing.^75^ Moreover, the antibacterial and regenerative properties of materials are of great importance. Therefore, the importance of innovative wound-healing materials, such as smart nanomaterials, cannot be overestimated. Smart nanomaterials, due to a combination of properties such as antimicrobial activity, drug delivery capabilities, modulation of cellular responses, or promotion of tissue regeneration, can respond to specific stimuli. This enables a more precise and controlled therapeutic approach than can be achieved with conventional materials.^76^ An aligned electrospun hydrogel fibre film integrated with mimetic PVP-IrNP nanozymes and AgNPs has been synthesised, providing a platform for high-performance wound-healing biomaterials (Table 1).^48^ This multifunctional smart material controls the concentration of NPs released through the self-degradation property of the hydrogel fibre film, making the application of this composite dressing *in vivo* safer. In addition, *in vitro* experiments confirmed that the integrated IrNP nanozymes could scavenge various ROS, protect cells against oxidative injury, while the AgNPs induced a pronounced antibacterial effect in the dressing. The NP-doped fibre film showed good biocompatibility and had no obvious inhibitory effect on cell proliferation activity, paving the way for the development of this innovative solution. However, accurate control of the release timing and concentration of the loaded NPs remains a challenge that needs to be addressed.

Thermoresponsive Pluronic®127 or hydroxypropyl methylcellulose gels incorporating AuNPs have been reported as promising platforms for AuNP formulations that exhibited prolonged and sustained effects compared to AuNP suspensions.^49,50^ The AuNP-loaded gels demonstrated effective antibacterial activity against *S. aureus* (*in vitro*) and promoted wound healing in burn-induced infected wounds in mice (*in vivo*).^49^ Furthermore, the optimal gelation temperatures, bio-adhesive forces and viscosities of these materials make them suitable for transdermal drug delivery systems. The biosynthesised Dicer subtract small interfering RNA (DsiRNA)-loaded AuNPs incorporated into a thermoresponsive Pluronic®127 gel have been developed to improve vascularisation by inhibiting PGT gene expression and preventing bacterial infection in a potential dressing that promotes the healing of diabetic wounds.^50^

Moreover, Ru-based compounds have been used to create hydrogels that facilitate wound healing by providing antibacterial protection, moisturising, and forming a protective layer on the wound. The main advantage of the Ru hydrogel over other hydrogels is that they are active under visible light.^77^

### 2.3 Anticancer therapy

In oncology, a wide range of metals have been investigated for their therapeutic potential. Gold nanoparticles are of particular interest as they easily penetrate cells, exert a strong cytotoxic effect, and can trigger long-term immune responses.^78^ Their biomedical activity is size-dependent, with smaller particles being more widely distributed in the body and exhibiting stronger cytotoxicity. Zhang *et al.*^58^ developed a new gold-based agent targeting gastric cancer cells and Richert *et al.*^59^ proposed Au(i)-phosphine complexes effective against ovarian cancer cells. Similarly, platinum compounds remain central to cancer therapy. Aromatic Pt(ii) complexes (pyrazoles/thiazoline and thiazine ligands), for example, show enhanced cytotoxicity against cervical and colon cancer cells.^27,60^ Pelka *et al.*^79^ found that PtNPs induce DNA strand breaks in human colon cancer cells (HT29) suggesting that they could be used as an alternative to cisplatin in the treatment of human colon cancer. However, while metallic platinum is generally safe, Pt complexes that interact with DNA are more reactive and may pose health risks. The toxicity of PtNPs also depends on particle size: extremely small particles (1 nm) can cause kidney damage and may accumulate in the liver and spleen, whereas slightly larger ones (8 nm) are significantly less harmful.^80^ Studies show that PtNPs cause hepatotoxicity and nephrotoxicity, but their carcinogenic effect has not been confirmed.^81^ Therefore, despite their effectiveness, the potential toxicity and long-term effects of platinum compounds remain a concern that requires further research and careful consideration in medical applications.

Pd complexes, though generally less potent than Pt, also display anticancer activity, and Pd-modified scaffolds are being explored to combine regenerative and anticancer properties. Abu-Dief *et al.*^61^ synthesised a new bioactive HNP–Pd complex (HNP = 1-(pyrimidin-2-yliminomethyl)-naphthalene-2-ol) which inhibited cancer cell growth and showed significant antimicrobial activity, although it also had significant toxicity. In turn, Rh and Ir complexes show selectivity toward cancer cells, with some Ir(iii) organometallic complexes surpassing cisplatin in potency against ovarian, cervical, and melanoma cancers.^82^ Adhikari *et al.*^83^ synthesised half-sandwich d^6^ metal complexes (Ir and Rh) containing 2-substituted-1,8-napthyridine ligands with unexpected binding modes. The change in the chelating ligand of a cytotoxic half-sandwich Ir(iii) complex was shown to affect the reactivity and selectivity of the organometallic Ir(iii) complexes. Moreover, as presented by Sadeghi *et al.*,^84^ a graphene-based aptasensor with RhNPs can be used in the detection and treatment of the HER2-ECD oncomarker, with higher efficiency, sensitivity and stability than the non-additive sensors.

Due to Ru cytocompatibility and efficient cross-linking, its compounds are attractive for drug delivery against liver, oral, renal, and lung cancers. They often improve selectivity and potency when combined with proteins such as albumin.^85,86^ Osmium complexes are equally noteworthy, as Os(ii)-arene complexes exhibit stronger cytotoxic activity against breast, colorectal, liver, kidney, and melanoma cells than the chemotherapeutics currently in use.^87–89^ For example, replacing Ru with the isostructural but chemically distinct Os in the organometallic protein kinase inhibitor (anticancer) scaffold (Ru → Os) does not significantly alter its biological activity. Os complexes combine anticancer with antimicrobial effects, making them promising dual-function agents.^89^

Taken together, these findings suggest that noble metals and PGMs are not only versatile anticancer agents but also promising alternatives to existing Pt-based drugs.

### 2.4 Dentistry

In dentistry, metals have played a long-established role. Au has traditionally been used in crowns, inlays, and bridges. Most dental high noble alloys are typically composed of at least 70% Au, Pt, and Pd, often accompanied by Ag. Together, these metals enhance corrosion resistance and biocompatibility, and provide antibacterial effects.^45,90^ In addition, for patients with contact allergies, noble alloys are now the most viable option.

Ag itself has a long history in dental applications, ranging from amalgam fillings to modern silver-doped hydroxyapatite (Ag HAP). The presence of Ag helps to prevent infections around implants thanks to its antibacterial and anti-inflammatory properties.^91^ Pt and Pd (*e.g.* as Au–Ag–Cu–Pd alloys) are also valuable in prosthodontics: Pt for crowns, bridges, and ceramic alloys, and Pd as part of multi-metal alloys for inlays and crowns.

An important role of Pt is to modify dental alloys to improve the bonding of porcelain to Ti frameworks, which remains challenging due to the formation of a passive TiO_2_ layer when the titanium is exposed to oxygen during porcelain firing. Ti-6Al-4V alloys, for example, modified with Pt or Ti/Pt films fabricated by additive manufacturing, have been shown to significantly enhance the Ti–porcelain bond. This demonstrates the feasibility of using Pt films on Ti-6Al-4V to achieve improved dental materials.^92^

Although Rh coatings for orthodontic NiTi wires have been reported to maintain the hardness of the material, they have also been found to increase corrosion when in contact with probiotics, due to greater roughness and friction compared to uncovered NiTi.^62,64^ A contrary, protective, effect of the Rh coating was observed on stainless steel archwires against the effects of NaF mouthwash. However, the positive effect of such a coating was attributed rather to Au presence in the noble alloy.^63^

Thus, dentistry remains one of the most consistent and practical fields of application for these metals.

### 2.5 Implants, scaffolds, and cardiac applications

AuNPs are vital for the regeneration of bone, cardiac, and neural tissues because they improve scaffold performance, enhance adhesion, and act as a tissue adhesive. The addition of AuNPs to polymeric nanofibrous scaffolds increases their stability and conductivity, while collagen/nanocrystalline cellulose/chitosan hydrogels loaded with AuNPs (collagen/CNCs/CS-Au) are characterised by improved mechanical strength and degradation resistance of the scaffolds.^41,52^ Different shapes, sizes and concentrations of AuNPs determine different physical properties (*e.g.* Au nanocages are more attractive for the controlled drug release than the Au nanoshells) and cytotoxicity (the smaller nanoparticle size, the higher cytotoxicity). Hence, Au can be widely used to improve electrical signalling, cell adhesion and mechanical properties of scaffolds and as tissue adhesives to connect the designed tissue patches to organs.^52^ This also makes them particularly useful for heart and neural tissue regeneration. Au nanospheres, rods and wires in the form of microwrinkles on polymer cantilevers helped cardiomyocytes grow in a manner similar to that observed in native cardiac tissues. The presence of AuNPs also improved conductivity in cardiac patches during cell-to-cell interactions. From a biomimetic engineering standpoint, such an Au-wrinkled device can stimulate cardiomyocyte maturation and enable accurate monitoring of drug cardiotoxicity.^65^

The antimicrobial and antiviral field relies heavily on Ag in various forms (metallic Ag, salts, complexes, NPs in the form of plates, rods, cubes, spheres), well known for its broad-spectrum activity against bacteria, viruses, fungi, and even algae. AgNPs disrupt cell walls and bind DNA and RNA, blocking replication. For this reason, they are incorporated into sutures, or coatings for implants. Ag plays a complementary role in preventing infection: it is applied as bactericidal coatings on titanium alloys and polymer implants such as polyetheretherketone (PEEK), and is also integrated into bioactive glasses and HAP, making orthopaedic and dental implants less susceptible to bacterial colonisation.^93,94^ The incorporation of graphene-Ag (rGO-Ag) into electrospun polyurethane scaffolds significantly improved conductivity, mechanical properties and, subsequently, cardiogenic differentiation potential. This makes the material a suitable candidate for cardiac tissue engineering.^46^ However, Ag health effects remain debated: metallic silver is biologically inert, but silver compounds may be carcinogenic, and high exposure can lead to argyria.^95^

Ru has found a niche in TE as well: it catalyses photo-cross-linking in fibrin-based scaffolds, stiffening engineered tissues without causing toxicity. It should also be highlighted that, according to Furrer *et al.*,^96^ Rh is not recommended in orthopaedic implant alloys. Although Rh is extremely corrosion resistant, it has poor mechanical properties; it is not recommended for bone plates.

Pd is increasingly studied in bone scaffolds, where its presence boosts antibacterial protection while maintaining strength. Meanwhile, Pt is a well-established material for pacemakers, defibrillators, surgical instruments and other implants thanks to its mechanical strength, corrosion resistance and biocompatibility. Stents made from an alloy containing 33% Pt, 18% Cr, 37% Fe, 9% Ni, 3% Mo and a trace amount of Mn have been considered an improved alternative to base metal stents due to their greater corrosion resistance, stability, and biocompatibility. The greater strength of the Pt alloy also enables manufacturers to reduce strut thickness without compromising the mechanical properties or radiopacity, which provides enhanced X-ray visualisation.^45^

Together, these metals bridge the gap between durable implant technology and regenerative approaches.

## 3 Rare earth elements (REEs)

The REEs, important for the medical applications, include two scandiums (scandium (Sc) and yttrium (Y)) and all of the lanthanides (Lns). Some examples of the applications of these metals in TE divided into light-lanthanide, heavy-lanthanide and scandium-based materials are presented in Tables 2–4.

**Table 2:** Light lanthanide-based materials in tissue engineering^a^

Application	Metal and material characteristics	Ref.
Bone regeneration & dentistry	**Lanthanum**	109, 111 and 197
	• La(iii) in OCP scaffolds → osteogenesis promotion, immunomodulation, bone regeneration	
	• La-doped HAP/CS magnetic scaffolds → stem cell adhesion and proliferation, bone tissue ingrowth	
	• La-doped BG/CS scaffolds → osteogenic and angiogenic differentiation, vascularization	
	**Cerium**	154, 198 and 199
	• CeO_2_ NPs (PCL/gelatin scaffolds) → osteogenic differentiation, good biocompatibility	
	• CeO_*x*_ coatings on TiO_2_ → improved cell adhesion, osseointegration, HAP maturation	
	• Ce-stabilized zirconia → bioactive, strong bonding to HAP.	
	**Samarium**	120, 138 and 200
	• Sm-HAP/PPy/titania biocomposites → antimicrobial, anticorrosion, cytocompatible scaffolds	
	• Dopped Sm-HAP → antibacterial activity against *E. coli, S. aureus, C. albicans*	
	• Nano HAP doped with Li, Eu(iii) and Sm(iii) → osteogenic activity, apoptosis inhibition, theranostic potential	
	**Neodymium**: Fe_3_O_4_NPs, Nd magnets in recapitulating the architecture of the native tissues → formation of microstructured scaffolds, VEGF/osteopontin expression, miniaturized tissue building blocks	201
Wound healing and skin repair	**Cerium**: CeO_2_NPs in ADM scaffolds → antioxidant activity, improved cell growth, enhanced collagen and tensile strength	97
	**Lanthanum**: La_2_O_3_NPs in collagen sponges → collagen stabilization, enhanced fibroblast proliferation, accelerated tissue repair	202
	**Neodymium**: Nd–Ca–Si glasses → photothermal and fluorescence thermometry properties, burn tissue repair, potential in PTT cancer therapy	203
	**Praseodymium**	143 and 204
	• Pr_2_O_3_NPs with collagen → endothelial and fibroblast cell activation, high cytocompatibility and hemocompatibility	
	• Collagen reinforced with Pr-cobaltiteNPs → angiogenesis promotion, thermal stability, collagen stabilization	

a BMSCs – bone mesenchymal stem cells, FAP – fluorapatite, HAP – hydroxyapatite, NP – nanoparticles, NT – nanotubes, PCL – poly(ε-caprolactone), PLA – polylactic acid, CS – chitosan scaffold, BG – bioglass.

**Table 3:** Heavy lanthanide-based materials in tissue engineering^a^

Application	Metal and material characteristics	Ref.
Bone regeneration	**Europium**: Eu-doped calcium polyphosphate → improved bone ingrowth, adhesion, mechanical strength, inhibition of bone resorption	3, 122, 205 and 206
	**Gadolinium**	112 and 207–209
	• Gd-HAP → adsorbs more serum proteins, increases cell viability, better cellular biocompatibility compared to La-HAP	
	• GdNPs-HAP, gelatine sponge with adipose-derived stem cells (ADSCs) → s good biocompatibility, possibility of chondrogenic cell differentiation, potential in repair cartilage defects	
	• GdPO_4_/chitosan scaffolds → promote osteogenic differentiation of rBMSCs by activating the Smad/Runx2 signalling pathway and increased osteogenic activity	
	• Gd-doped mesoporous calcium silicate in porous scaffolds → activate wnt/β-catenin signalling pathway, excellent cell proliferation and osteogenic differentiation capacities, stimulation of collagen deposition and new bone formation	
	**Terbium**: Apatite fibrils addition of Tb to collagen/apatite scaffolds → luminescent nanocomposites, osteogenic promotion	210
	**Thulium**: Tm(iii) and Yb(iii) ions doped silica–calcia glass nanopowder → HAP-like surface, bone regeneration and defect repair	211
	**Ytterbium**: Yb-doped HAP nanorod arrays deposit in magnetic (SrFe_12_O_19_) in chitosan scaffolds → osteogenesis & vascularization *via* VEGFA and BMP-2/Smad pathway	212
Bone and bioimaging	**Erbium**: Er(iii) : Tb(iii) co-doped BG powders → photoluminescent biomaterials for TE, drug release, imaging	213
Bone defect implants	**Dysprosium**: In Dy–ZrO_2_ stainless steel 316L alloys → Dy addition improved strength, corrosion resistance, biocompatibility	214
Wound healing	**Terbium**: Tb(OH)_3_ and Eu(OH)_3_ nanorods → bioimaging, magnetic/optical activity, wound dressing potential	144
	**Europium**: PCL incorporated with europium hydroxide nanorods (EHN) scaffolds → vascularization, hemocompatibility, endothelial adhesion	215
Cardiac	**Europium**: Eu–tannic acid bioactive materials → antioxidation, angiogenesis, myocardial repair	103
Tumour imaging, targeted therapy, and repair	**Holmium**: Hydrogel Ho-containing BG, poloxamer 407 → osteoblast proliferation & osteosarcoma cell death, injectable cancer therapy	216
	**Europium**: Eu–Gd-Si–Ca glass decorated by FAAL molecule → high biocompatibility, inhibition of tumour recurrence, enhancement of tissue regeneration, and imaging of skin tumour tissue	217
Imaging and diagnostic applications	**Ytterbium**: Eu, Gd, Yb bioactive glass → photoluminescence, HAP formation, broad TE potential	218

a BMSCs – bone mesenchymal stem cells, FAP – fluorapatite, HAP – hydroxyapatite, NP – nanoparticles, NT – nanotubes, PCL – poly(ε-caprolactone), PLA – polylactic acid, SBF – simulated body fluid, CS – chitosan scaffold, BG – bioglass.

**Table 4:** Scandium-based materials in tissue engineering

Application	Characteristics	Ref.
Dentistry	**Scandium**: Sc-doped phosphate-glasses (Sc-PBGs) with addition of CaO, Na_2_O → antibacterial and anticancer properties	140
Orthopaedic	**Scandium**: 0.1Sc or 0.1Y addition to alloys Ti–24Nb–38Zr–2Mo → mechanical properties Sc shows better properties than Y → material strength and desirable biological characteristics	219
Scaffolds enhanced angiogenesis	**Yttrium**: PCL with Y_2_O_3_ NPs → improves the angiogenesis by upregulating the expression of VEGF and EGFR.	220
Bone regeneration	**Yttrium**	139 and 221
	• Y (1–15%) doped HAP → antibacterial and antifungal properties (*S. aureus*, *P. aeruginosa*, *E. coli*, and *C. albicans*), promising material for bone cement engineering with a potential bioactivity	
	• HAP/Y_2_O_3_/GO (graphene oxide) nanocomposite is → biocompatible, improves hardness (from 2.5 for pure HAP to 2.9 GPa), and antibacterial activity	

**Fig. 3:**
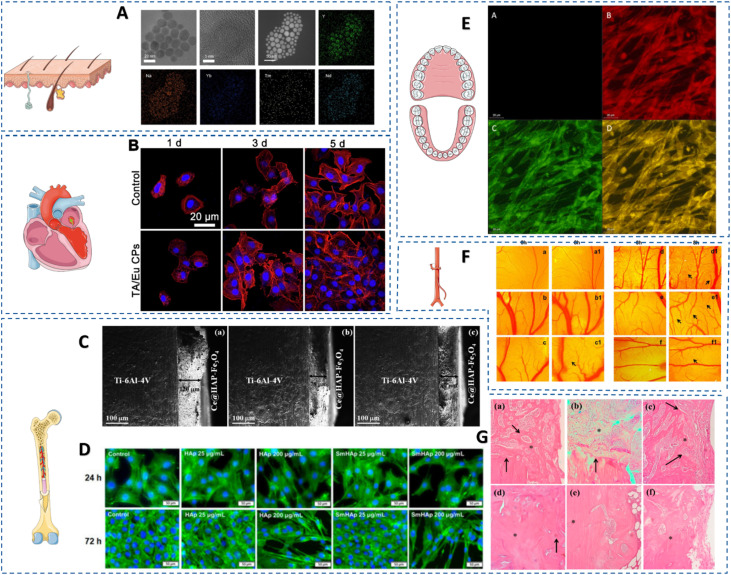
Representative applications of REE-based materials in tissue engineering. (A) NaYF_4_:Yb^3+^,Tm^3+^@NaYF_4_:Nd^3+^,Yb^3+^ + GelMa (UC-YT@NY + GelMa) hydrogel hybrids to *in vivo* wound healing: HRTEM images of UC-YT@NY and a large view of the area image of upconversion nanocrystals. Reprinted from ref. 102. (B) Polyphenol-europium assembly coordination complex with enhanced antioxidation and angiogenesis for myocardial infarction treatment: confocal laser scanning microscopy images of HUVECs treated with TA/Eu CPs for 1, 3 and 5 days. Reprinted from ref. 103. (C) Cerium-incorporated HAP/magnetite nanocomposite coatings with bone regeneration and osteosarcoma potential: cross-sectional view of Ce-HAP/Fe_3_O_4_ composite coating on Ti-6Al-4V obtained at 2000 rpm (a), 3000 rpm (b), 4000 rpm (c). Reprinted from ref. 104. (D). Samarium-doped HAP-biomimetic nanoceramics for bone regeneration: fluorescent images of the organization of the F-actin cytoskeleton in MC3T3-E1 preosteoblasts exposed to HAP and SmHAP at concentrations of 25 and 200 μg mL^−1^ for 24 and 72 h. Actin filaments are stained with Alexa Fluor 488 phalloidin (green) and the cell nuclei with Hoechst (blue). Scale bar, 50 μm. Reprinted from ref. 105. (E) Gadolinium and selenium doped bioactive glass surface coating for dental implants: biocompatibility analysis of developed implant coating using confocal microscope, (A) blank, (B) rhodamine B staining, (C) acridine orange staining, (D) combination staining. Reprinted from ref. 106. (F) Electrospun polycaprolactone (PCL) scaffolds embedded with europium hydroxide nanorods (EHNs) with enhanced vascularization and cell proliferation for tissue engineering applications: *in vivo* angiogenesis assay using chick embryo model. The chick embryo incubated with PCL scaffolds containing (a and a1) untreated; (b and b1) neat PCL (0% EHNs); (c andc1) PCL-EHNs-0.25; (d and d1) PCL-EHNs-0.5; (e and e1) PCL-EHNs-1 & (f and f1) PCL-EHNs-2. Reprinted from ref. 107. (G) Cerium-containing HAP in bone repair: trabecular bone neoformation with immature aspect, with variable number of osteoblasts and osteocytes (arrows). 15 days: (a) = SC group. (b) = CC group and (c) = Ce-HA-OVX group; 30 days: (d) = SC group, (e) = CC group and (f) = Ce-HA-OVX group. Reprinted from ref. 108. (Free images from Smart Servier Medical Art, website: https://www.smart.servier.com).

Due to the similar electron configuration (4f^*n*^5d^1^6s^2^ or 4f^*n*+1^6s^2^) of these metals, the Lns share similar properties, and typically occur in the third oxidation state. Some can also form ions of different valence (II or IV), which is associated with obtaining a more stable 4f^0^, 4f^7^, or 4f^14^ electron configuration (*e.g.*, Ce^4+^, Tb^4+^, Yb^2+^, Eu^2+^). Moreover, the outer 5s significantly shield the 4f electron, resulting in unique optical properties, such as extended lifetime emission or narrow band widths with strong fluorescence emission through photoluminescence.^97^ Without these metals, it is difficult to imagine diagnostics,^98^ TE and regenerative medicine.^99^ Although La, Ce and Gd are the most frequently mentioned, but all Lns have medical applications (Fig. 3). Significant progress in biological applications of Lns began in 2011, with the discovery of their incorporation into bacterial methanol dehydrogenase.^100^ Trace amounts of Lns, like La, Ce, Pr, Nd, Sm, Gd, Tb, Ho, Tm, Yb, and Lu, which occur naturally in organisms, play an important role in regulating stem cell differentiation, metabolism, and tissue regeneration.^101^ However, the impact of REE when incorporated into bioactive materials is ambiguous. Depending on the dose, their influence on living organisms can be either positive or negative. Nevertheless, the mechanism by which REEs incorporated into bone regeneration have a positive impact is not yet understood. Furthermore, there are insufficient data on the safety of using REE biomaterials in living organisms. Therefore, the manufacturing of REE biomaterials still faces challenges in achieving their theoretical therapeutic benefits.

### 3.1 Bone regeneration

Bone regeneration is a complex process involving coordinated cellular activities such as osteoblast differentiation, mineralisation, and vascular integration. Among the rare earth elements (REEs), several lanthanides and related metals have emerged as promising modulators of bone formation due to their ability to enhance osteogenesis, influence scaffold properties, and regulate stem cell behaviour.

Lanthanides can substitute for calcium, the primary mineral component of bone, in HAP, raising concerns about bone mineralization and cellular function. This substitution occurs because the ionic radii of lanthanides (Ln^3+^), particularly La^3+^, and Ca^2+^ are similar, allowing La^3+^ to integrate into the bone matrix. Despite the strength and implications of this exchange reaction are complex and not fully understood, the potential benefits of La-substituted HAP and La-doped bioactive glass in bone repair have been investigated due to their enhanced osteogenesis, antibacterial properties, and mechanical stability. La-doped mesoporous bioglass/chitosan composite scaffolds have been shown to promote osteogenesis, suggesting their potential in bone tissue engineering applications.^109,110^ La, similarly to Gd and Sc, promotes osteogenic differentiation of BMSCs *via* the Wnt/β-catenin pathway, enhancing bone formation.^111^ Lanthanum substitution in HAP has been reported to influence the mechanical properties of the material, potentially enhancing its suitability as a bone graft substitute. It should be noted that the potential toxicity of La ions is dose-dependent.^112^ Important observations on the cellular biocompatibility of nano-La-HAP compared to nano-Gd-HAP showed differences in cell viability. It was observed that Gd-HAP exhibits superior properties and shows higher protein adsorption capacity than La-HAP, which mitigates their cytotoxicity by reducing its cellular uptake. This research indicates that the presence of Gd in controlled doses reduces the toxicity of HAP, facilitating the development of a highly bioactive HAP. Its high magnetic moment enables dual functionality serving both as an MRI contrast agent and a bioactive component in scaffolds targeting osteosarcoma.^113^ For instance, the gadolinium and silicon co-doped HAP/poly(lactic-*co*-glycolic acid) composite (1.5Gd&Si-HAP/PLGA) showed greater osteoinductive properties than of undoped HAP. The bone repair scaffold prepared using 1.5Gd&Si-HAP/PLGA composite materials has a broad application prospects in bone TE, with Gd additionally playing a role as an MRI contrast agent *in vivo*.^114^

Thus, while La ions can substitute for calcium in bone tissue, leading to potential toxicity concerns, the controlled incorporation of La-derived compounds in bone repair materials has been shown to offer benefits such as enhanced osteogenesis, antibacterial properties, and improved mechanical stability. At low concentrations, La can have a positive effect on the bone remodelling cycle and could be used to treat bone density disorders. However, higher concentrations could be detrimental. Therefore, careful control of La concentration in biomedical applications is essential to harness its benefits while minimising risks. For example, cytotoxicity studies carried out on human embryonic kidney (HEK) cell lines revealed that 5% La-incorporated apatite can be regarded as the safe limit concentration of lanthanum in this material.^115^

Cerium (Ce) compounds also contribute to bone regeneration by promoting osteoblast activity and reducing osteoclast numbers, thus favourably influencing the bone remodelling cycle.^116^ The redox cycling between Ce^3+^ and Ce^4+^ endows cerium oxide nanoparticles (CeONPs, known as nanoceria) with antioxidant properties that protect cells from oxidative stress, potentially enhancing cell survival during early stages of regeneration. Adjusting the Ce^4+^/Ce^3+^ ratio changes biomaterial properties, making it a strategic approach for designing orthopaedic/dental implants with a favourable immune response.^117^ These features confirm that Ce_*x*_ONPs can be incorporated into polymer scaffolds or bioactive glasses and used as an angiogenic structure in TE.^118^ Samarium (Sm), and europium (Eu)further support bone growth through distinct mechanisms. Sm improves osteoblastic performance by reducing the number of osteoclasts.^119^ As a result, it has found applications in medicine, including TE (Table 2), particularly in bone growth where the concentration of Sm(iii) ions in the HAP lattice is crucial.^120^ Similarly to Gd, combining the optical properties of Eu with its ability to restore bone tissue, Eu-doped HAP scaffolds exhibit osteogenic effects, and scaffold adsorption itself could be monitored by the luminescent properties of Eu^3+^, indicating treatment progress.^121^ Incorporating Eu_2_O_3_ into the CaO–SiO_2_–P_2_O_5_–MgO–CaF_2_ BG system improved the crystallisation of the material, but inhibited the formation of fluorapatite. This resulted in a scaffold with satisfactory porosity, good mechanical properties and excellent biocompatibility, which promotes cell adhesion, proliferation and differentiation.^122^ Moreover, Eu is the most reactive lanthanide that has been studied in recent decades for its potential to enhance angiogenesis. Eu is known for its osteoangiogenic effects and its ability to promote endothelial cell proliferation and vascularisation (angiogenic effects),^118,123^ Example applications of Eu, as well as other heavy lanthanides, in TE are presented in Table 3. Dysprosium (Dy)- and ytterbium (Yb)-doped HAP or bioglass systems also promote cell adhesion and differentiation while enabling optical tracking due to their intrinsic luminescence, offering real-time assessment of regeneration progress. Dy-doped HAP or BG-based materials can be used for luminescent imaging, as an alternative to organic fluorophores and quantum dots,^124,125^ taking advantage of its two characteristic emission bands at 480 nm and 575 nm due to ^4^F_9/2_ to ^6^H_15/2_ (blue) and ^4^F_9/2_ to ^6^H_13/2_ (yellow). Due to the similar size of the ionic radius, Yb^3+^ ions can easily replace Ca^2+^ ions in HAP, which promotes the use of Yb in bone regeneration.

Erbium (Er), while primarily known for laser applications, also exhibits biocompatibility and indirect support for bone regeneration when doped into HAP nanoparticles or strontium-based matrices (Er^−^doped HAP NPs,^126^ Sr-based Er-doped HAP^127^). PrNPs also show promise in applications for bone TE and implantology.^97^

Sc(iii) ions have a valence state similar to lanthanide ions and an ionic radius similar to Li(i) ions (0.0745 and 0.076 nm, respectively), and therefore have a high potential to regulate cell activity. Scandium alloys with Mg exhibit high mechanical strength and corrosion resistance, and can be used as bone implants in the form of metallic scaffolds (*e.g.* Sc is able to transform Mg from *hcp* to *bcc* structure and reduce the content of impurities (Fe, Ni, Cu)).^24,128^ The highly offensive biological properties of Sc(iii) ions are not without significance. ScCl_3_ affects the proliferation of mesenchymal stem cells, promotes osteogenesis, and influences osteogenic and adipogenic differentiation by activating the Wnt/β-catenin signalling pathway.^129^ The properties of scandium mentioned above and outlined in Table 4 have determined its use in TE. Furthermore, Y can be used as a stabiliser for zirconia, and applied in orthopaedic and dental applications due to its favourable mechanical properties, bioinert nature and aesthetics, which are particularly important in dentistry for the levelling of dental cavities.^130^ These materials are corrosion-resistant because of the transformation toughening mechanism (high chemical stability). Moreover, Y exhibits anti-inflammatory properties by regulating the expression of genes associated with apoptosis and inflammation.^24^

In addition, the beneficial effects of a static neodymium magnetic field on bone regeneration and wound healing have been demonstrated.^131–133^ Due to the piezoelectric properties of bones, external physical stimulation can accelerate the process of bone formation after injury. Gujjalapudi *et al.*^131^ found that the implant stability coefficient and tissue response following implant placement were significantly higher under the influence of a magnetic field than on the non-magnetic side. This positive correlation between magnetic field and osteointegration has been confirmed by other researchers in cases of various bone defects.^132^

Bone regeneration is a primary objective in the design of implantable TE constructs. However, the prevention of microbial infection is equally critical for ensuring long-term clinical success. Several REEs not only promote osteogenesis but also exhibit intrinsic antibacterial and antifungal properties, enabling the development of infection-resistant biomaterials. For example, La incorporation into bioceramic scaffolds confers antibacterial activity, important for preventing postoperative infections in bone repair procedures.^134^ Similarly, cerium oxide nanoparticles (CeONPs) exhibit bactericidal effects against human lung pathogens and other bacterial strains, especially when combined with Pd doping (Pd-dop-CeO_2_).^135^ Additionally, Pr exhibits antibacterial and antifungal activity^136^ due to the change in the surface permeability of a prokaryotic organism.^137^ Sm,^138^ Y^139^ and Sc^140^ also display antimicrobial effects across various microbial strains. For example, Sc-based pharmaceuticals are explored for antibiotic development due to their ability to regulate microbial cell activity.^141^

Although REEs, especially lanthanides, are being incorporated into bioactive scaffolds for bone regeneration, where they regulate stem cell differentiation and enhance tissue repair, the mechanisms of their interaction and long-term safety are still under investigation.

### 3.2 Wound healing and soft tissue regeneration

Wound healing involves coordinated phases of inflammation, proliferation, and remodelling. Certain lanthanides have been found to accelerate soft tissue repair by modulating immune responses, stimulating granulation tissue formation, enhancing re-epithelialisation and enhancing angiogenesis.

Praseodymium (Pr) is a relatively new area of research in TE. Despite its limited use, the biocompatibility and bioabsorbability of Pr in alloy form warrant further investigation. Doping titanium nitride coatings with Pr not only enhances corrosion resistance, but also reduces the rate of haemolysis, suggesting the usefulness of such coatings in cardiovascular applications.^142^ Praseodymium oxide NPs promote soft tissue regeneration by enhancing angiogenesis and accelerating tissue repair through VE-Cadherin expression in the wound microenvironment.^143^ Terbium (Tb) hydroxide nanorods control the capacity to induce ROS production and NO formation, both mediators of angiogenic signalling, and supporting their use in wound healing as well as cardiovascular and ischaemic diseases.^144^ Europium (Eu) has demonstrated beneficial effects on collagen deposition and re-epithelialization in models of chronic skin injury, further validating its role in in wound-healing and skin-regeneration.^145^ When incorporated into hydrogels or bioactive glasses, Eu provides both structural support and biological cues to promote tissue restoration.

Yttrium oxide (Y_2_O_3_, also known as yttria) has antioxidant and radical scavenging properties, which also indicate its high potential to enhance cell proliferation and the angiogenic properties of TE scaffolds.^139^ Additionally, Y regulates apoptosis- and inflammation-related gene expression, contributing to balanced immune responses during tissue repair. Moreover, Tb hydroxide nanorods control the capacity to induce ROS production, NO formation, and angiogenesis and can be used in wound healing as well as cardiovascular and ischaemic diseases.^144^

Additionally, as mentioned in 3.1 Section, a static magnetic field sourced by neodymium permanent magnets has been reported to improve wound healing and alleviate diabetic complications.^75,133^ It has been suggested that a magnetic field can affect the ROS levels and oxidative stress in cells by altering the electron spin states of metabolic intermediates in living organisms, and thus influencing biochemical reactions. In consequence, a reduced ROS level has been reported to decrease nuclear NRF2 translocation and MDA levels, thereby promoting wound healing in diabetic mice and reducing liver lipid accumulation and renal defects.^75^ Moreover, as with Nd, the magnetic properties of Sm, such as in samarium cobalt (SmCo_5_), induce the proliferation of human umbilical cord-derived mesenchymal stem cells (hUC-MSCs) and can be used to promote the propagation of stem cells for clinical therapy.^146^ The study also showed that samarium in combination with cerium (SmCeO_2_ nanoconjugates) triggered endothelial cell proliferation and induced blood vessel growth in the chicken embryo. Sm compounds (6-{2-[2-(2-methoxy-ethoxy)-ethoxy]-ethoxy}hexyl)triethoxysilane conjugated with SmCeO_2_ NPs are proposed for the treatment of cardiovascular, ischaemic and ocular diseases.^147^

Moreover, Duraipandy and Kiran^148^ pointed out that Nd can be used to induce stem cell differentiation by detecting ROS induction (angiogenic response through the VEGFR-2 pathway). They emphasised the crucial role of the structure of NdNPs in modulating the angiogenic process by promoting endothelial cell growth and proliferation *via* cell–cell attachment and redox signalling pathways. It opens up the possibility of using REE biocompatible NPs as a new therapeutic agent in wound healing. Cuboid and rod-shaped NdNPs activated the proangiogenic factors (VE-cadherin, HIF-1α, VEGF and VEGFR-2) more effectively than spherical NPs to facilitate the angiogenic process. Based on these findings, novel biocompatible therapeutics can be developed to regulate disease pathophysiology using REE NPs.

These findings indicate that specific REEs can be strategically integrated into wound care materials to simultaneously address infection control, and contributing directly or indirectly to angiogenesis *via* redox modulation, gene expression regulation, and scaffold-mediated signalling enhancement.

### 3.3 Anticancer therapy and tumor inhibition

Several REE exhibit anticancer properties through mechanisms involving ROS generation, photothermal effects, radiation therapy, and targeted drug delivery.

Mesoporous silica nanostructures coated with La/Sr/Mn oxide NPs show potential for cancer treatment by magnetic hyperthermia,^149^ while Vinothini *et al.*^150^ proposed the encapsulation of the anticancer drug Paclitaxel and lanthanum oxide NPs in a polymeric micelle. Their work indicates that lanthanum oxide affects the production of reactive oxygen species (ROS), thereby inhibiting the growth of cancer cells undergoing chemotherapy. The recognised high potential of the research resulted in the search for simple and green methods for the synthesis of lanthanum NPs. For example, a safe phytosynthesis method of La_2_O_3_ NPs using *C. guianensis* abul leaf extract as reducing agent was reported^151^ and the resulting NPs exhibited activity against the proliferation of MCF-7 breast cancer cells. However, the concentration of lanthanum in biological systems is crucial, with low concentrations providing beneficial effects and high concentrations being toxic to healthy cells and tissues (as mentioned also in Section 3.1).^152^ Proper concentration management can harness the potential of lanthanum in medical applications. Some of the most recent applications are listed in Table 2.

CeONPs display dual functionality in cancer therapy: they scavenge ROS in normal cells (protective effect), yet generate cytotoxic radicals in tumour environments under specific conditions. For example, PEG-supported bimetallic Pd-doped CeONPs (Pd-dop-CeO_2_) enhance therapeutic efficacy against bacterial pathogens and human lung cancer cells.^135^ CeONPs have anti-inflammatory properties, scavenging reactive species *in vitro* and *in vivo.*^153^ Furthermore, nanoceria can be used to reduce cytokine levels and provide cellular protection, including neural, retinal, hepatic, and cardiac cells against oxidative stress (due to redox cycling between cerium in the fourth and third oxidation states: Ce^4+^ → Ce^3+^) and inflammatory responses.^154^ CeONPs are also effective in cancer treatment, including lung cancer by producing free radicals and damaging membranes, which is associated with reduced cell viability^135^ and ovarian cancer by complexing NPs with folic acid.^155^

Praseodymium (Pr), erbium (Er), and ytterbium (Yb) all demonstrate anticancer activity against multiple cell lines, including Vero, MCF-7 (breast cancer), and HeLa (cervical cancer).^156^ The mechanism likely involves mitochondrial dysfunction and ROS induction. Dy is used in medicine mainly for imaging and to a lesser extent for treatment. The radioactive isotope ^165^Dy is used in the treatment of injured joints^157^ and in cancer therapy.^158^

Gd, known for its large magnetic moment, long electron relaxation time, and high relaxivity, is commonly used as a contrast agent in MRI or CT, *i.e.* for tumour analysis.^98^ Beyond its typical diagnostic utility, Gd-doped polydopamine NPs demonstrate therapeutic potential as bone-targeting platforms that inhibit osteoclast formation and suppress tumour-induced bone resorption. Under near-infrared irradiation, these NPs exhibit photothermal properties capable of effectively inhibiting osteosarcoma growth and associated osteolysis, a promising approach for treating metastatic bone cancers.^113,114^ Due to the dual function of Gd in tissue engineering is also mentioned in Section 3.1.

Lutetium (Lu)-based radiopharmaceuticals such as ^177^Lu-EDTMP and ^177^Lu-DOTMP are clinically used to treat painful bone metastases due to their combined β^−^ emission for therapy and γ radiation for imaging. At the same time, the use of ^177^Lu isotope is beneficial in the post-chemotherapy of metastatic castration-resistant prostate cancer.^159^ Additionally, Sm is also proposed as a drug for the treatment of various types of cancer (lung, prostate, breast, osteosarcoma, *etc.*) and pain therapy for bone metastases.^138^

Thus, REEs offer a diverse arsenal for oncological applications ranging from direct cytotoxicity to image-guided therapy, emphasizing their growing importance in cancer TE strategies.

### 3.4 Imaging and diagnostic applications

The unique optical and magnetic properties of many REEs enable the non-invasive monitoring of tissue development, cellular dynamics, and disease progression. An ultra-wide Stokes shift in the luminescence, variable magnetism and potentially tuneable properties of LnNPs have been highlighted as crucial properties for the development of new applications for image-guided surgery.

Gd, as mentioned above, is commonly applied as a contrast agent in MRI and CT imaging attributed to its strong magnetic moment, prolonged electron relaxation, and high relaxivity.^98^ Dy-doped HAP or bioglass materials emit characteristic blue (480 nm) and yellow (575 nm) luminescence bands due to ^4^F_9/2_ → ^6^H_15/2_ and ^4^F_9/2_ → ^6^H_13/2_ transitions, enabling their use as luminescent probes in place of organic fluorophores or quantum dots.^124,125^ While Er-heavily doped NPs under 808 nm excitation maximally increase absorption and optimise the energy of ^4^I_13/2_ → ^4^I_15/2_ transition.^160^ Also, Nd is primarily used in medical applications because of its optical properties (BW-I ∼800 and 880 nm and BW-II ∼1050 and 1320 nm), which make it useful for tissue imaging and temperature detection.^161^Dy has also been shown to exhibit paramagnetic behaviour due to its high magnetic moment. This phenomenon has been exploited by Tesch *et al.*^162^ in the development of luminescent and magnetic HAPs by doping with Eu (to impart photoluminescent properties) and Dy. Moreover, terbium (Tb), like a number of other Lns, due to its optical properties is being exploited mainly for bioimaging in diagnosis and disease control, with emission spectra in the blue (^5^D_3_–^7^F_J_) and green (^5^D_4_–^7^F_J_) regions. Tb(iii)-doped HA has fluorescent properties and the potential to be used in drug delivery, therapy, and *in vivo* bioimaging,^163^ similar to Tb-doped mesoporous silica.^164^ Additionally, the fluorescent properties of Yb are used in probes, such as β-NaGdF_4_:Yb/Er NPs for stem cell tracking, with simultaneous proliferation and differentiation.^165^

Er occurs naturally in living organisms, for example in the bones of the ribs. Therefore it appears to be a good material for biomedical applications, in particular imaging. Erbium (Er)-based nanocrystals are also employed for short-wave infrared bioimaging,^166^ Er–Yb Schiff base metal complexes as fluorescent staining dyes^167^ or Er-doped near-infrared II (NIR-II) luminescent NPs.^160,168^ A study on the use of near-infrared-IIb (NIR IIb, 1500–1700 nm) fluorescence from Er-based lanthanide NPs for imaging-guided surgery of orthotopic glioma reported that NaErF_4_:2.5%Ce@NaYbF_4_(0.9 nm)@NaLuF_4_DCNPs with dye-brush polymer (Dye-BP) are the optimal down-conversion nanoparticles (DCNPs) for significantly enhancing fluorescence in an aqueous solution, due to their excellent energy-cascaded downconversion properties, compared to NaErF_4_ NPs. These highly bright NPs were modified with a tumour-targeting angiopep-2 peptide and were delivered efficiently to the glioma for diagnosis. The size of the glioma tumour measured from the width of the fluorescence profile closely matched that measured from the T_2_-weighted MRI images, demonstrating the great potential of NIR IIb fluorescence-guided surgery for tumours.^160^

Holmium (Ho) and thulium (Tm), though primarily utilised in medical lasers, also contribute to diagnostic modalities. Ho is used in medicine as a source of radionuclides for diagnostic (SPECT, MRI and CT) and therapeutic applications, mainly as medical lasers. It has been used for years in urology to treat the prostate (Holmium Laser Enucleation of the Prostate (HoLEP)). In addition, the feasibility of using an ultrafast all-fibre holmium laser system on muscle tissue has been demonstrated.^169^ Due to its radiation properties, ^166^Ho has also found application in brachytherapy, for example as a sol–gel derived bioactive glass containing holmium.^170^ This takes advantage of the fact that the Ho emits high-energy β-rays, but with limited penetration into body tissues in the 8.4 mm range, limiting side effects on healthy cells adjacent to the treated area. Tm, like the other Lns mentioned, is used in medical lasers. For example, the Tm fibre laser (TFL) is more effective and safer than the Ho:YAG laser for urological procedure,^171^ including lithotripsy and soft tissue ablation^172^ and endoscopic enucleation of the prostate.^173^ Furthermore, Eu compounds are used in bioanalysis due to luminescent properties.^174^ Promethium (Pm) is the only element in the lanthanide series that has no stable isotopes. The ^147^Pm isotope is the most widely used, particularly in long-life nuclear batteries. Pm has limited medical applications, including beta therapy for lumbosacral radiculitis, PET imaging, and therapy for hair loss.^175^ In addition, Lu compounds, due to its ability to emit not only β but also γ radiation, Lu is used for bone imaging.^176^ Isotopes of Sc have also been used as radioactive tracers,^177^ and in complexes with other isotopes, *e.g.* Sc-^18^F ternary complexes in PET imaging^178^ and radiopharmaceuticals.^179^ Although Yb does not occur naturally in the human body, it has several medical applications. The ^169^Yb isotope, is used for brachytherapy due to its 32-half-life and 93 keV gamma-ray energy. Yb also has luminescent properties^180^ Yb- and Er-co-doped NPs allow simultaneous tracking and assessment of stem cell differentiation states *via* upconversion luminescence, facilitating non-invasive monitoring in regenerative settings.^165^

These elements provide powerful tools for real-time, non-destructive evaluation of engineered tissues and pathological conditions.

### 3.5 Drug delivery systems

Controlled release of therapeutic agents is vital in TE for localised treatment with minimal off-target effects. Several REEs facilitate smart drug delivery through magnetic responsiveness, luminescent tracking, or stimuli-responsive behaviour. Lanthanide compounds have been proposed for drug delivery, for example, due to their unique paramagnetic properties,^181^ they can be used for the controlled release of drugs through magnetic field control. For instance, mesoporous silica nanostructures coated with La/Sr/Mn oxide nanoparticles enable cancer treatment *via* magnetic hyperthermia.^149^ Encapsulation of paclitaxel and La_2_O_3_NPs in polymeric micelles enhances chemotherapeutic efficacy through ROS-mediated cytotoxicity.^150^

Apart from their osteogenic activity described in Section 3.1, La, Ce, Pr, Eu, Nd, and Dy doped on mesoporous bioactive glass (MBG) nanospheres can effectively operate as a pH- and time-dependent release system to improve the anticancer efficacy of doxorubicin and reduce its side effects. As a result, the developed REE/MBG nanospheres (containing 1 mol% RE ions) might be useful as versatile implantable delivery systems for clinical bone repair, with the possibility of treating bone defects caused by cancer.^182^ Ce-based carriers, are also effective in cancer treatment, including lung cancer by producing free radicals and damaging membranes, which is associated with reduced cell viability^135^ and ovarian cancer by complexing NPs with folic acid.^155^ Furthermore, Eu compounds have been used in drug delivery systems. For example, Eu-doped fluorapatite nanorods have been applied to release doxorubicin for the treatment of melanoma A375 cells.^183^

Chitosan-Eu-doped HAP beads were synthesised to reduce the initial burst release of ciprofloxacin.^184^ Thanks to a combination of enhanced fluorescence and the ability to release drugs, Eu-doped beads can be used to monitor the release and distribution of antibiotics. The beads' intrinsic fluorescence enables real-time bioimaging and targeted drug delivery. This multifaceted approach to design offers tremendous opportunities for creating customised systems that ensure controlled drug release and maintain therapeutic levels, representing a significant advance in biomedical applications.

Also, Gd has found various applications in drug delivery. For example, Gd-doped polydopamine NPs have been proposed as scaffolds for a bone-targeting peptide (aspartic acid) to inhibit osteoclast formation and bone destruction, as well as photothermal properties. Thus, the Gd NPs supported by near-infrared lasso radiation effectively inhibited tumour growth and bone resorption and can be used to treat osteosarcoma and related osteolysis. However, Gd can cause nephrogenic systemic fibrosis in patients with severely impaired kidney function.^112^

### 3.6 Medical lasers and biomaterial modification

Medical lasers based on REE-doped crystals are indispensable tools in surgery and biomaterial processing. Their precise ablation capabilities enable minimally invasive procedures and microstructuring of scaffolds.

Holmium (Ho):YAG lasers are widely used in urology for prostate enucleation (HoLEP) and lithotripsy.^172^ Thulium (Tm)-doped fibre lasers offer superior precision and safety compared to Ho:YAG in urological procedures such as endoscopic enucleation and soft tissue ablation.^173^ Tm-doped fibre lasers can also modify the topography of temperature-sensitive biomaterials creating pores, microchannels, or foams.^185^ The results obtained indicate potential uses of Tm-doped fibre lasers in cell and TE. However, recent research (*ex vivo* porcine model) indicates that protective goggles should be worn when using Tm lasers, due to the risk of ocular injury.^186^ Although this is currently the main direction of Tm application in medicine, the literature points to the search for alternative solutions in TE, including as an additive to BG.^187^

Moreover, the Nd:YAG (Nd-doped Yttrium Aluminum Garnet) laser can be a support in TE, due to its ability to clean the contaminated implant surfaces for periodontal tissue, increase the activity of osteoblasts, be very effective in accelerating for the deposition of minerals,^188^ and be used in cataract surgery.^189^ Near-infrared irradiation at 800–880 nm and 1050–1320 nm allows deep–tissue interaction for imaging and thermal therapy.^161^ It is also worth mentioning the Nd laser and the possibility of using it to modify biological materials (such as collagen, hyaluronic acid, and chitosan) with it by irradiation to create materials with high biomedical potential.^190^ Er:YAG lasers enable effective bone ablation,^191^ while Cr:Er:YSGG lasers enable laser-induced microstructuring and TE, *i.e.*, immature tooth model.^192^

### 3.7 Radiation shielding and protection

In therapeutic contexts involving ionising radiation (*e.g.*, brachytherapy, diagnostic imaging), protecting the surrounding healthy tissue is of the highest importance. Certain REE-doped bioactive glasses offer inherent shielding capabilities.

Glass powders containing Er(iii) and Tb(iii) can be used as radiation shielding materials in TE applications, making them suitable for use in TE constructs exposed to radiotherapy environments.^193^ Similarly, Dy-containing silicate-based BGs exhibit luminescent imaging potential alongside protective functions.^180^ Gd-containing silicate-based BG can also suppress gamma radiation.^194^ Er is also used in radiation shielding materials, such as Er(iii)- and Tb(iii)-containing silicate-based bioactive glass powders,^195^ sodium zinc borate-Er_2_O_3_ glasses,^196^ and lasers.

These materials represent multifunctional platforms that integrate structural support, biological activity, and radioprotection, particularly valuable in oncological TE applications.

## 4 Key findings

The key findings emerging from the literature review highlight new future directions for the applications of noble metals and REEs in TE and medical applications:

• Multifunctionality of new biomaterials: particularly REE biomaterials combine biocompatibility, fluorescence upconversion, and drug release ability. These properties make them ideal for use in bone or soft tissue engineering, when coupled with diagnostic and treatment applications.

• Smart nanomaterials: innovative nanomaterials have led to the development of smart biomaterials that, in responsive way, enhance osteogenic, angiogenic, immunomodulatory, and antimicrobial properties, facilitating better wound healing, bone regeneration and implant integration.

• HAP doped with REE: new data on the influence of incorporating various REEs into HAP on biocompatibility, cytotoxicity, and other properties. This line of research contributes to the identification of the mechanisms through which some REEs impact tissues.

• Surface modifications: Pt, Au, and Ag are used to modify implant surfaces, improving biocompatibility and antibacterial properties. This approach aims to create multifunctional implants that can better integrate with biological tissues and reduce infection risks.

## 5 Challenges and future perspective

**Fig. 4:**
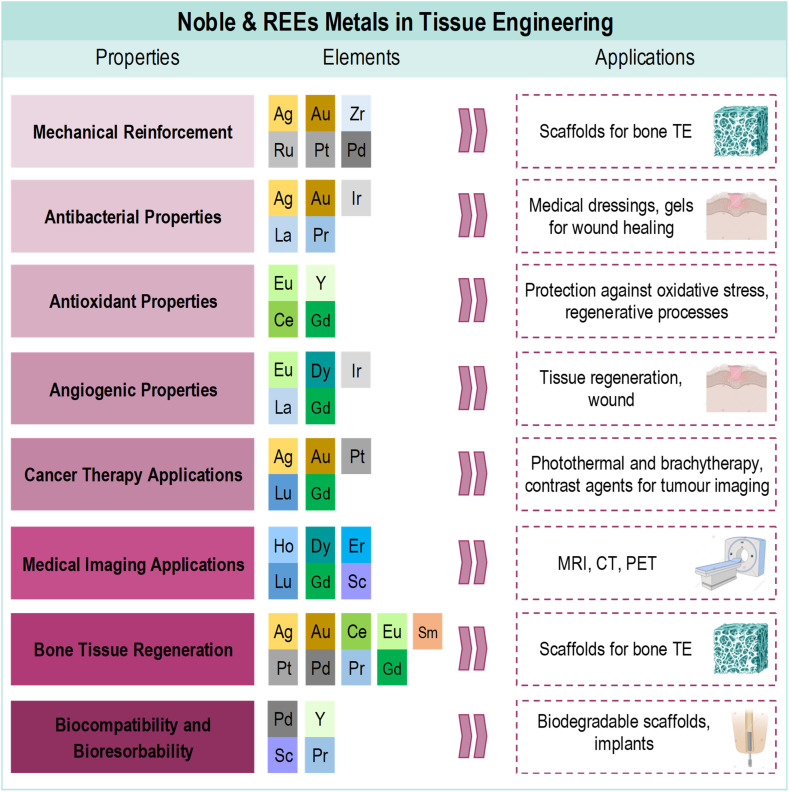
Key properties and applications of noble and REEs metals in tissue engineering (free images from bioRender website: https://www.biorender.com).

Due to their unique chemical and physical properties, noble metals and REEs can play an important role in, for example, improving the mechanical properties of the scaffolds or increasing the antibacterial properties (Fig. 4 and Tables 1–4). Scaffolds with the addition of metallic NPs such as Ru, Pd, Au, Pt, Ag, La, Ce, Eu, Gd, Er are used as carriers for the bone cells cultivation in bone TE. For example, the combination of chitosan (biopolymer) with the supporting properties of Gd can enhance bone regeneration and wound healing. Furthermore, many bioactive metals (*e.g.* Au, Ag, Ir, La, and Pr) have antibacterial properties, making them suitable for incorporation into medical dressings and gels to facilitate wound healing and skin regeneration. However, the impact of these metals on the human organism has not been fully investigated.

The long-term effects of metals on human health remain a significant challenge. Although many of these materials have exhibited promising results *in vitro* and in animal models, their precise mechanisms of action remain to be fully elucidated, and there is an absence of substantial human testing. Future research should prioritise both mechanistic studies to better understand their interactions with human tissues and long-term their interaction into their potential toxicity and adverse effects to ensure that their clinical applications are based on a solid scientific foundation. Additionally, achieving consistent and targeted therapeutic outcomes with noble metals and REEs is challenging. Although various noble metals and REEs have shown potential in biomedical applications, metals such as Au, Ag, and Gd have demonstrated particular effectiveness in enhancing bone regeneration and wound healing. However, further investigation is required to elucidate their precise mechanisms of action and optimal dosages. The development of targeted delivery systems and precise dosage optimisation will be crucial for enhancing therapeutic efficacy while minimising cytotoxicity. Further research is needed to elucidate the biodistribution, and cellular interactions, as well as the long-term effects of these metals. Safe and effective dosage parameters must also be established through pharmacokinetic modelling and controlled clinical studies.

It is also important to consider the scalability and cost-effective manufacturing processes when transitioning from laboratory research to clinical applications. The synthesis of materials based on noble metals and REEs can be complex and expensive, which hinders the widespread adoption of these materials. Future research should focus on developing innovative and sustainable manufacturing techniques that can produce these materials on a large scale without compromising quality, thereby making them more accessible for clinical use.

In conclusion, noble metals and REEs demonstrate promising potential in TE, applications, particularly in enhancing scaffold properties, promoting biocompatibility, and supporting tissue regeneration, as evidenced by recent studies. However, there are still several challenges to overcome, such as determining the long-term effects of these materials on human health. Further research is needed to investigate their biocompatibility, safety, and therapeutic efficacy. Continued research and innovation in this area is essential to realise the full potential of these bioactive metals in improving human health.

## Conflicts of interest

There are no conflicts to declare.

## Data Availability

No primary research results, software or code have been included and no new data were generated or analysed as part of this review.
